# Clinical trauma severity of indoor and outdoor injurious falls requiring emergency medical service response

**DOI:** 10.1186/s40621-024-00517-1

**Published:** 2024-08-09

**Authors:** Kathryn G. Burford, Nicole G. Itzkowitz, Remle P. Crowe, Henry E. Wang, Alexander X. Lo, Andrew G. Rundle

**Affiliations:** 1 Department of Environmental Health Sciences, Columbia University Mailman School of Public Health, 722 West 168 th Street, Room 1616, New York, NY 10032 USA; 2 Department of Epidemiology, Columbia University Mailman School of Public Health, New York, NY 10032 USA; 3 ESO Solutions LLC, Austin, TX USA; 4 Department of Emergency Medicine, Wexner Medical Center, Ohio State University, Columbus, OH USA; 5 Department of Emergency Medicine, Northwestern University Feinberg School of Medicine, Chicago, IL USA; 6 Center for Health Services & Outcomes Research, Northwestern University Feinberg School of Medicine, Chicago, IL USA

**Keywords:** Falls, Injuries, Outdoor falls, Surveillance

## Abstract

**Background:**

Injurious falls represent a significant public health burden. Research and policies have primarily focused on falls occurring indoors despite evidence that outdoor falls account for 47–58% of all falls requiring some medical attention. This study described the clinical trauma severity of indoor versus outdoor injurious falls requiring Emergency Medical Services (EMS) response.

**Methods:**

Using the 2019 National Emergency Medical Services Information System (NEMSIS) dataset, we identified the location of patients injured from falls that required EMS response. We classified injury severity using (1) the Revised Trauma Score for Triage (T-RTS): ≤ 11 indicated the need for transport to a Trauma Center; (2) Glasgow Coma Scale (GCS): ≤ 8 and 9–12 indicated severe and moderate neurologic injury; and (3) patient clinical acuity by EMS: Dead, Critical, Emergent, Low.

**Results:**

Of 1,854,909 encounters for patients with injurious falls, the vast majority occurred indoors (*n* = 1,596,860) compared to outdoors (*n* = 152,994). For patients who fell indoors vs outdoors on streets or sidewalks, the proportions were comparable for moderate or severe GCS scores (3.0% vs 3.9%), T-RTS scores indicating need for transport to a Trauma Center (5.2% vs 5.9%) and EMS acuity rated as Emergent or Critical (27.7% vs 27.1%). Injurious falls were more severe among male patients compared to females and males injured by falling on streets or sidewalks had higher percentages for moderate or severe GCS scores (5.2% vs 1.9%) and T-RTS scores indicating the need for transport to a Trauma Center (7.3% vs 3.9%) compared to falling indoors. Young and middle-aged patients who fell on streets or sidewalks had higher proportions for a T-RTS score indicating the need for Trauma Center care compared to those in this subgroup who fell indoors. Yet older patients injured by falling indoors were more likely to have a T-RTS score indicating the need for transport to a Trauma Center than older patients who fell on streets or sidewalks.

**Conclusions:**

There was a similar proportion of patients with severe injurious falls that occurred indoors and outdoors on streets or sidewalks. These findings suggest the need to determine outdoor environmental risks for outdoor falls to support location-specific interventions.

## Introduction

Falls represent an enormous global public health burden associated with significant disability and mortality, with a worldwide age-standardized incidence of 2238 falls per 100,000 persons per year in 2017, over 16.6 million years of life lost, and an average loss of 4% of one’s full health status from one fall (James et al. 2020). The US Centers for Disease Control and Prevention (CDC) reported 7.9 million unintentional injurious falls in 2019, associated with 131.5 billion USD in medical costs (Injury Center CDC 2023). Although falls affect all ages, the burden of falls in the US is disproportionately borne by older persons, for whom falls are the leading cause of disability and functional decline (James et al. 2020; Panel on Prevention of Falls in Older Persons 2011).

Research and policy attention has been primarily devoted to falls occurring indoors (Panel on Prevention of Falls in Older Persons 2011; American Geriatrics Society, British geriatrics society, and American academy of orthopaedic surgeons panel on falls prevention 2001) despite reports that among community-dwelling adults, outdoor falls account for 47–58% of falls requiring at least some medical attention (Li et al. 2006; Timsina et al. 2017). The CDC’s Web-Based Injury Statistics Query and Reporting System (WISQARS), the Behavioral Risk Factor Surveillance System (BRFSS) and the National Health Interview Survey (NHIS) are primary public health surveillance systems in the US for fall-related injuries (Injury Center CDC 2023; Timsina et al. 2017; Moreland et al. 2020). However, none of these systems routinely provide data on the locations in which falls occur. To improve the surveillance of outdoor falls, Rundle et al. (2023) developed a methodology to identify injurious falls by indoor versus outdoor location using Emergency Medical Services (EMS) clinical and administrative data from the National Emergency Medical Services Information System (NEMSIS) (Rundle et al. 2023). Among the 1,854,909 injuries from falls that required an EMS response in 2019, 129,408 of these fall injuries were identified as occurring outdoors on streets and sidewalks, a number which is 70% higher than the number of pedestrians reportedly injured by automobiles (Li et al. 2006; Timsina et al. 2017; National Highway Traffic Safety Administration 2019).

While establishing surveillance methods is a critical first step towards developing interventions to reduce outdoor falls, epidemiological data are still needed to understand the public health and clinical burden of outdoor falls. Specifically, improved understanding of the clinical severity for fall injuries is critical to determine the short- and long-term burdens of 1) morbidities and disability among individuals (Stewart Williams et al. 2015) and 2) health care utilization needs of different populations (Eliacin et al. 2021; Korley et al. 2016), particularly in light of potential differences across sociodemographic groups (Chun Fat et al. 2019; Sharma et al. 2018). In the immediate setting following an injury, clinical severity scoring tools have been recommended, including the Revised Trauma Score for Triage (T-RTS) and Glasgow Coma Scale (GCS), to help guide on-scene EMS to determine the severity of the injury and optimal care response for the individual (Champion et al. 1989; Newgard et al. 2022).

There are very few available studies that compare the severity of indoor and outdoor falls, with the focus of these being among the older adult population (Bath and Morgan 1999; Chippendale et al. 2017; Kelsey et al. 2010, 2012; Kim 2016; Lee 2021; O’Loughlin et al. 1994). Chippendale et al.’s (2017) study of older U.S. trauma center patients who sustained outdoor falls had a higher frequency of open wounds and head injuries compared to indoor falls, but no significant difference on the injury severity score (ISS). Kim (2016) found that outdoor falls led to a higher proportion of head and neck injuries than indoor falls among older emergency department patients across 20 hospitals in Korea. Jung et al.’s study (2018) indicated that the likelihood of severe injury, as determined by level of care, from outdoor falls in older adults was higher in men compared to women. Overall, these studies were limited by examining only individuals admitted to a hospital, and none examined a national sample. Furthermore, assessment of outcomes and measures of injury severity varied across these studies limiting the ability to compare findings.

National surveillance data comparing indoor and outdoor injurious falls are almost non-existent, yet critical to the development of person-centered, community-specific programs and policies to prevent serious falls. Data from EMS responses on the clinical trauma severity and level of care for indoor and outdoor injurious falls could be particularly informative from a healthcare resource perspective. Here we use 2019 national U.S. EMS data to describe the clinical trauma severity of indoor versus outdoor injurious falls, and to describe these patterns by patient demographic characteristics.

## Methods

### Study design

This cross-sectional study of EMS records from the 2019 National Emergency Medical Services Information System (NEMSIS) Public-Release Research Dataset included 1,854,909 occurrences of falls requiring EMS response across US states and territories (Rundle et al. 2023).

### Data source

NEMSIS is the national system to collect and standardize data from EMS agencies across the US that is administered by the National Highway Traffic Safety Administration (NHSTA) Office for Emergency Medical Services. The NEMSIS data are public, de-identified, and HIPAA exempt data released by the University of Utah, as such further Institutional Review Board (IRB) review was not requested (Dawson 2006; Ehlers et al. 2023). The use of NEMSIS to identify falls and locations of falls have been previously described and includes a robust approach to identifying overall injurious falls and to identifying falls for which syncope (heat-related and non-heat related syncope) and heat illness were contributing factors Rundle et al. (2023). EMS data entry into NEMSIS must abide by the standards set forth by the NHTSA Office of EMS and outlined in the NEMSIS data dictionary (https://nemsis.org/media/nemsis_v3/release-3.5.0/DataDictionary/PDFHTML/EMSDEMSTATE/index.html) (NEMSIS 2024).

### Study variables and inclusion criteria

Detailed methods for inclusion criteria and coding of fall locations (indoor, outdoor – not on street or sidewalk, outdoor – on street or sidewalk, indoor/outdoor unclear), patient demographic variables, and on-scene clinical measures (e.g. patient acuity) can be found in Rundle et al. (2023). Briefly, we used NEMSIS variables ePatient 13, ePatient 15 and eSituation 13 to define patient sex (male, female) and age groups (0–20, 21–30, 31–40, 41–50, 51–60, 61–65, 66–70, 71–75, 76–80, 81–85, 86–90 and 91 + years). Falls with EMS notations of seizures have been removed from the analyses. NEMSIS data includes the patient’s clinical acuity, rated by EMS, which is classified as: Dead Without Resuscitation Efforts, Critical, Emergent, Low, Unknown. NEMSIS data were also used to calculate the Revised Trauma Score for Triage (T-RTS) and the Glasgow Coma Scale (GCS). The T-RTS, GCS and patient acuity data were used to characterize the severity of the injuries as observed by the EMS clinician on scene. When the tests used to calculate the GCS and the T-RTS were administered multiple times for a patient, the mean of all administrations were used. Sensitivity analyses were repeated using the first, maximum (or best), and minimum (or worst) scores for GCS and T-RTS.

### Revised trauma score for triage (T-RTS)

The Revised Trauma score for Triage to a Trauma Center (T-RTS) is a modified version of the original Trauma Score, that is more reliable and excludes capillary refill and respiratory expansion which are more difficult to assess in the field by EMS (Champion et al. 1989). The T-RTS can be used by EMS to make decisions on trauma care based on the severity of patient injuries (Lichtveld et al. 2008). For each patient, we calculated the mean T-RTS by summing the average value for GCS, systolic blood pressure, and respiratory rate. Champion and colleagues evaluated T-RTS cut-points based upon survival probabilities to create decision rules for patients to be triaged to a trauma center (Champion et al. 1989). Decision Rule 2 was used for the present study to categorize the T-RTS score into: ≤ 11, indicated need for immediate transport to a Trauma Center designated hospital; > 11 does not. As blood pressure may be artificially lowered by anti-hypertensive medications, the use of which differs by age group, the validity of the T-RTS may vary by age. Therefore, we decided a priori to also assess GCS alone as a second measure of injury severity that is independent of blood pressure.

### Glasgow coma scale (GCS)

The Glasgow Coma Scale (GCS) is a well-established measure of neurological status used for risk stratification in acute neurosurgical or traumatic injuries; the scale ranges from 3 to 15 and is calculated by summing the values for eye opening, verbal response, and motor response for each patient. In contemporary clinical practice, the GCS is used to determine the risks of mortality and morbidity, and more urgently to guide acute clinical management. We calculated the mean Glasgow Coma Scale (GCS) by summing the average value for eye opening, verbal response, and motor response for each patient. GCS ranges from 3 to 15, and for this analysis, we used the common GCS classifications for injury severity: severe, ≤ 8; moderate, 9–12; and minor, ≥ 13 (Jain and Iverson 2024).

### Statistical analyses

We conducted descriptive analyses for all EMS encounters for injurious falls by comparing the T-RTS, GCS, and patient acuity classifications by fall location, and further described these analyses by patient demographics. All results were interpreted among non-missing data. Due to the large size of this dataset, we did not include measures of statistical significance as even quite small differences in percentages reported within tables and cross-tables are statistically significant. Instead, we allow the readers to make interpretations based on practical relevance in the differences. We conducted all analyses in R statistical software (v4.3.1; R Core Team 2023).

## Results

Table 1 reports on the location of fall injuries by injury severity scores. In total 1,854,909 injuries from falls that required an EMS response were identified in the 2019 NEMSIS data. While the majority of falls occurred indoors (91%), among falls occurring outdoors, 85% occurred on streets and sidewalks. For patients who fell indoors compared to outdoors on streets or sidewalks, proportions were similar for moderate or severe GCS scores, T-RTS scores indicating need for transport to a Trauma Center and EMS acuity rated as Emergent or Critical. Patients injured by falling outdoors not on a street or sidewalk had a lower percentage for moderate or severe GCS scores and for T-RTS scores indicating the need for transport to a Trauma Center compared to percentages for those injured by falling outdoors on streets or sidewalks, but the reverse pattern was observed for patients injured by falls with an Emergent or Critical patient acuity.

**Table 1:** Descriptive statistics for EMS Encounters for Reported Location of Fall Injuries by Patient Acuity, GCS and T-RTS scores.^1^

	Overall		Indoor		Outdoor- street or sidewalk		Outdoor- not on street or Sidewalk		Indoor/outdoor unclear		Missing	
	N = 1854909		N = 1596860		N = 129408		N = 23586		N = 53700		N = 51355	
*Patient Acuity*												
Dead	1600	0.1%	1432	0.1%	92	0.1%	29	0.2%	32	0.1%	15	0.1%
Critical	40609	3.0%	35267	2.9%	3044	3.2%	890	4.8%	1073	4.2%	335	2.1%
Emergent	339764	24.8%	302119	24.8%	22522	23.9%	4840	26.3%	6955	27.2%	3328	21.0%
Low	991674	72.3%	880467	72.3%	68834	72.9%	12681	68.9%	17498	68.5%	12194	76.9%
Missing	481262	25.9%	377575	23.6%	34916	27.0%	5146	21.8%	28142	52.4%	35483	69.1%
*GCS*												
Severe	16508	1.0%	13753	0.9%	1614	1.4%	214	1.0%	613	1.2%	314	0.8%
Moderate	34914	2.0%	30227	2.0%	2922	2.5%	257	1.2%	848	1.7%	660	1.6%
Minor	1663581	97.0%	1439013	97.0%	114490	96.2%	20843	97.8%	49624	97.1%	39611	97.6%
Missing	139906	7.5%	113867	7.1%	10382	8.0%	2272	9.6%	2615	4.9%	10770	21.0%
*T-RTS*												
Need for transport to Trauma Center	84220	5.2%	72748	5.2%	6494	5.9%	849	4.4%	2408	5.0%	1721	4.5%
Does not	1528682	94.8%	1324148	94.8%	104366	94.1%	18250	95.6%	45534	95.0%	36384	95.5%
Missing	242007	13.0%	199964	12.5%	18548	14.3%	4487	19.0%	5758	10.7%	13250	25.8%

^1^GCS, Glasgow Comma Scale; T-RTS, Revised Trauma Score for Triage

Table 2 reports on the location of fall injuries by injury severity scores and by patient sex. A higher proportion of male patients had injuries rated as Critical or Emergent, had moderate to severe GCS scores and had T-RTS scores indicating the need for transport to a Trauma Center, than female patients. The proportions for male patients injured by falls who had moderate or severe GCS scores and T-RTS scores indicating the need for transport to a Trauma Center were also higher for outdoor falls on streets or sidewalks compared to indoor falls. While among female patients, the percentages for moderate or severe GCS scores and T-RTS scores necessitating care at a Trauma Center were more similar for falls on streets or sidewalks and indoors locations. Among male and female patients, the proportions for injurious falls for which patient clinical acuity was rated Critical or Emergent were similar for falls occurring on streets or sidewalks and for falls occurring indoors.

**Table 2:** Descriptive Statistics for EMS Encounters for Reported Location of Fall Injuries by Patient Acuity, GCS and T-RTS Scores, Categorized by Sex.^1^

	Overall		Indoor		Outdoor- street or sidewalk		Outdoor- not on street or sidewalk		Indoor/outdoor unclear		Missing	
	N = 1854909		N = 1596860		N = 129408		N = 23586		N = 53700		N = 51355	
*Acuity*												
Female												
Dead	611	0.1%	559	0.1%	36	0.1%	2	0.0%	6	0.0%	8	0.1%
Critical	18700	2.4%	16981	2.3%	930	2.4%	311	3.6%	340	2.8%	138	1.5%
Emergent	190691	24.1%	174488	24.1%	9105	23.0%	2115	24.4%	3153	25.6%	1830	20.0%
Low	582325	73.5%	530657	73.4%	29465	74.5%	6244	72.0%	8799	71.5%	7160	78.4%
Missing	275103	25.8%	223437	23.6%	14541	26.9%	2395	21.6%	14227	53.6%	20503	69.2%
Male												
Dead	983	0.2%	868	0.2%	56	0.1%	27	0.3%	26	0.2%	6	0.2%
Critical	21729	3.8%	18137	3.7%	2098	3.8%	572	5.9%	727	5.5%	195	3.8%
Emergent	147941	25.6%	126701	25.7%	13331	24.4%	2703	27.9%	3762	28.6%	1444	25.6%
Low	406624	70.4%	347588	70.5%	39171	71.7%	6402	66.0%	8626	65.6%	4837	70.4%
Missing	202742	26.0%	151433	23.5%	20051	26.8%	2709	21.8%	13809	51.2%	14740	69.5%
Missing Sex	7460	0.4%	6011	0.4%	624	0.5%	106	0.4%	225	0.4%	494	1.0%
*GCS*												
Female												
Severe	5916	0.6%	5295	0.6%	301	0.6%	48	0.5%	160	0.6%	112	0.5%
Moderate	16757	1.7%	15398	1.7%	659	1.3%	75	0.7%	281	1.1%	344	1.5%
Mild	966752	97.7%	860083	97.7%	48921	98.2%	9908	98.8%	24860	98.3%	22980	98.1%
Missing	78005	7.3%	65346	6.9%	4196	7.8%	1036	9.4%	1224	4.6%	6203	20.9%
Male												
Severe	10540	1.5%	8416	1.4%	1309	1.9%	165	1.5%	451	1.8%	199	1.2%
Moderate	18041	2.5%	14732	2.5%	2250	3.3%	182	1.6%	565	2.2%	312	1.9%
Mild	691717	96.0%	574772	96.1%	65211	94.8%	10862	96.9%	24697	96.0%	16275	97.0%
Missing	59721	7.7%	46807	7.3%	5937	7.9%	1204	9.7%	1337	4.9%	4436	20.9%
Missing Sex	7460	0.4%	6011	0.4%	624	0.5%	106	0.4%	225	0.4%	494	1.0%
*T-RTS*												
Female												
Need for transport to Trauma Center	40132	4.3%	36,222	4.3%	1811	3.9%	310	3.4%	929	3.9%	860	3.9%
Does not	895796	95.7%	797768	95.7%	45008	96.1%	8748	96.6%	22944	96.1%	21328	96.1%
Missing	131502	12.3%	112132	11.9%	7258	13.4%	2009	18.2%	2652	10.0%	7451	25.1%
Male												
Need for transport to Trauma Center	43823	6.5%	36305	6.5%	4659	7.3%	539	5.4%	1471	6.1%	849	5.4%
Does not	628624	93.5%	522887	93.5%	59069	92.7%	9444	94.6%	22451	93.9%	14773	94.6%
Missing	107572	13.8%	85535	13.3%	10979	14.7%	2430	19.6%	3028	11.2%	5600	26.4%
Missing Sex	7460	0.4%	6011	0.4%	624	0.5%	106	0.4%	225	0.4%	494	1.0%

^1^GCS, Glasgow Comma Scale; T-RTS, Revised Trauma Score for Triage

Tables 3, 4 and 5 report on the injury severity measures by the location of fall and by patient age. Young and middle-aged patients (≤ 60 years) who were injured by falls on streets and sidewalks had higher proportions for T-RTS scores indicating the need for transport to a Trauma Center compared to young and middle-aged patients injured by falls occurring indoors. However, older patients (> 60 years) who were injured by falling indoors had higher proportions for T-RTS scores indicating the need for transport to a Trauma Center than older adults who fell on streets or sidewalks. Similar patterns were observed for patients who had moderate or severe GCS scores for falls. Results were essentially the same whether the GCS and T-RTS score were calculated using the mean, or the first, maximum, or minimum scores.

**Table 3:** Descriptive Statistics for EMS Encounters for Reported Location of Fall Injuries by GCS, Categorized by Age.^1^

	Overall		Indoor		Outdoor- street or sidewalk		Outdoor- not on street or sidewalk		Indoor/outdoor unclear		Missing	
	*N* = 1854909		*N* = 1596860		*N* = 129408		*N* = 23586		*N* = 53700		*N* = 51355	
*0–20*												
Severe	777	0.7%	576	0.7%	106	1.3%	23	0.4%	61	0.7%	11	0.5%
Moderate	1671	1.6%	1322	1.6%	193	2.4%	49	0.8%	77	0.9%	30	1.3%
Mild	104427	97.7%	79877	97.7%	7818	96.3%	6089	98.8%	8310	98.4%	2333	98.3%
Missing	11264	9.5%	8554	9.5%	881	9.8%	674	9.9%	613	6.8%	542	18.6%
*21–30*												
Severe	1006	1.5%	625	1.4%	272	2.3%	27	1.4%	65	1.5%	15	1.0%
Moderate	1637	2.5%	1079	2.3%	403	3.5%	40	2.1%	92	2.1%	23	1.6%
Mild	62932	96.0%	44496	96.3%	10947	94.2%	1849	96.5%	4199	96.4%	1441	97.4%
Missing	5377	7.6%	3582	7.2%	1037	8.2%	219	10.3%	252	5.5%	277	15.8%
*31–40*												
Severe	1205	1.6%	829	1.5%	255	2.1%	26	1.4%	73	1.7%	22	1.4%
Moderate	1994	2.6%	1349	2.4%	495	4.0%	31	1.7%	88	2.1%	31	2.0%
Mild	72806	95.8%	53734	96.1%	11678	94.0%	1805	96.9%	4053	96.2%	1536	96.7%
Missing	6004	7.3%	4198	7.0%	1104	8.2%	181	8.9%	213	4.8%	308	16.2%
*41–50*												
Severe	1404	1.5%	1037	1.4%	236	1.7%	35	1.9%	75	1.7%	21	1.1%
Moderate	2334	2.4%	1690	2.3%	472	3.5%	20	1.1%	116	2.6%	36	1.8%
Mild	93031	96.1%	72137	96.4%	12839	94.8%	1807	97.0%	4343	95.8%	1905	97.1%
Missing	7444	7.1%	5452	6.8%	1174	8.0%	195	9.5%	203	4.3%	420	17.6%
*51–60*												
Severe	2271	1.2%	1851	1.2%	262	1.2%	31	1.2%	89	1.2%	38	1.0%
Moderate	3990	2.1%	3086	2.1%	639	2.9%	43	1.7%	150	2.1%	72	1.9%
Mild	180072	96.6%	145551	96.7%	21454	96.0%	2477	97.1%	6935	96.7%	3655	97.1%
Missing	14168	7.1%	10975	6.8%	1767	7.3%	311	10.9%	301	4.0%	814	17.8%
*61–65*												
Severe	1417	1.1%	1203	1.1%	113	1.0%	13	0.9%	61	1.4%	27	1.0%
Moderate	2384	1.9%	2016	1.9%	243	2.1%	17	1.1%	72	1.7%	36	1.4%
Mild	124861	97.0%	105365	97.0%	11367	97.0%	1487	98.0%	4100	96.9%	2542	97.6%
Missing	9724	7.0%	7922	6.8%	924	7.3%	146	8.8%	173	3.9%	559	17.7%
*66–70*												
Severe	1497	1.0%	1304	1.0%	99	1.0%	17	1.2%	54	1.4%	23	0.8%
Moderate	2553	1.8%	2320	1.8%	123	1.3%	14	1.0%	44	1.1%	52	1.8%
Mild	140898	97.2%	123198	97.1%	9534	97.7%	1420	97.9%	3860	97.5%	2886	97.5%
Missing	11168	7.2%	9360	6.9%	808	7.6%	146	9.1%	183	4.4%	671	18.5%
*71–75*												
Severe	1493	0.9%	1337	0.9%	84	1.0%	16	1.2%	38	1.0%	18	0.5%
Moderate	3062	1.8%	2856	1.9%	99	1.1%	10	0.7%	46	1.2%	51	1.4%
Mild	163497	97.3%	146552	97.2%	8464	97.9%	1351	98.1%	3610	97.7%	3520	98.1%
Missing	13134	7.2%	11195	6.9%	689	7.4%	104	7.0%	154	4.0%	992	21.7%
*76–80*												
Severe	1578	0.9%	1451	0.9%	51	0.7%	13	1.2%	27	0.8%	36	0.8%
Moderate	3471	1.9%	3257	2.0%	91	1.2%	7	0.6%	47	1.4%	69	1.6%
Mild	178017	97.2%	162117	97.2%	7397	98.1%	1072	98.2%	3233	97.8%	4198	97.6%
Missing	14677	7.4%	12491	7.0%	647	7.9%	107	8.9%	175	5.0%	1257	22.6%
*81–85*												
Severe	1386	0.7%	1273	0.7%	46	0.7%	3	0.4%	28	0.9%	36	0.7%
Moderate	3889	2.0%	3725	2.0%	37	0.6%	9	1.2%	40	1.3%	78	1.5%
Mild	192234	97.3%	177428	97.3%	6189	98.7%	741	98.4%	2956	97.8%	4920	97.7%
Missing	15326	7.2%	13146	6.7%	521	7.7%	68	8.3%	134	4.2%	1457	22.4%
*86–90*												
Severe	1281	0.7%	1211	0.7%	26	0.6%	3	0.7%	18	0.8%	23	0.4%
Moderate	3845	2.0%	3677	2.1%	45	1.1%	9	2.0%	39	1.7%	75	1.4%
Mild	185542	97.3%	173300	97.3%	4209	98.3%	446	97.4%	2299	97.6%	5288	98.2%
Missing	15636	7.6%	13391	7.0%	342	7.4%	59	11.4%	110	4.5%	1734	24.4%
*91 + *												
Severe	1034	0.6%	951	0.6%	25	1.1%	4	1.6%	18	1.0%	36	0.7%
Moderate	3890	2.3%	3714	2.4%	39	1.7%	5	1.9%	31	1.8%	101	1.9%
Mild	162219	97.1%	152813	97.0%	2242	97.2%	248	96.5%	1677	97.2%	5239	97.5%
Missing	13867	7.7%	11931	7.0%	187	7.5%	33	11.4%	56	3.1%	1660	23.6%
Missing Age	5515	0.3%	4346	0.3%	733	0.6%	86	0.4%	109	0.2%	241	0.5%

^1^GCS, Glasgow Comma Scale

**Table 4:** Descriptive Statistics for EMS Encounters for Reported Location of Fall Injuries by Patient Acuity, Categorized by Age

	Overall		Indoor		Outdoor- street or sidewalk		Outdoor- not on street or sidewalk		Indoor/outdoor unclear		Missing	
	*N* = 1854909		*N* = 1596860		*N* = 129408		*N* = 23586		*N* = 53700		*N* = 51355	
*0–20*												
Dead	51	0.1%	41	0.1%	5	0.1%	2	0.0%	2	0.0%	1	0.1%
Critical	2232	2.6%	1642	2.4%	220	3.3%	161	3.0%	190	3.5%	19	2.0%
Emergent	18631	21.5%	14482	21.2%	1369	20.7%	1256	23.4%	1326	24.3%	198	20.9%
Low	65689	75.9%	52049	76.3%	5021	75.9%	3960	73.6%	3931	72.1%	728	77.0%
Missing	31536	26.7%	22115	24.5%	2383	26.5%	1456	21.3%	3612	39.9%	1970	67.6%
*21–30*												
Dead	51	0.1%	35	0.1%	12	0.1%	3	0.2%	0	0.0%	1	0.2%
Critical	1802	3.5%	1244	3.3%	326	3.5%	102	6.2%	112	4.7%	18	3.2%
Emergent	12151	23.4%	8819	23.2%	2034	21.9%	455	27.6%	689	28.8%	154	27.3%
Low	37928	73.0%	27925	73.4%	6929	74.5%	1089	66.0%	1593	66.5%	392	69.4%
Missing	19020	26.8%	11769	23.6%	3360	26.5%	486	22.8%	2214	48.0%	1191	67.8%
*31–40*												
Dead	81	0.1%	51	0.1%	18	0.2%	5	0.3%	7	0.3%	0	0.0%
Critical	2224	3.7%	1685	3.7%	357	3.6%	65	4.1%	103	4.8%	14	2.2%
Emergent	14845	24.7%	11401	24.9%	2272	23.0%	415	26.3%	598	27.7%	159	25.0%
Low	42917	71.4%	32659	71.3%	7249	73.3%	1095	69.3%	1452	67.2%	462	72.8%
Missing	21942	26.8%	14314	23.8%	3636	26.9%	463	22.7%	2267	51.2%	1262	66.5%
*41–50*												
Dead	98	0.1%	77	0.1%	14	0.1%	5	0.3%	2	0.1%	0	0.0%
Critical	2866	3.7%	2250	3.7%	385	3.6%	91	5.8%	106	5.0%	34	4.3%
Emergent	19459	25.4%	15762	25.6%	2499	23.4%	417	26.5%	602	28.2%	179	22.7%
Low	54304	70.8%	43446	70.6%	7793	72.9%	1061	67.4%	1428	66.8%	576	73.0%
Missing	27486	26.4%	18781	23.4%	4030	27.4%	483	23.5%	2599	54.9%	1593	66.9%
*51–60*												
Dead	218	0.1%	194	0.2%	10	0.1%	6	0.3%	7	0.2%	1	0.1%
Critical	5277	3.6%	4399	3.6%	542	3.1%	143	6.4%	150	4.6%	43	2.8%
Emergent	37159	25.0%	31178	25.2%	4064	23.0%	639	28.7%	919	27.9%	359	23.3%
Low	105835	71.3%	87952	71.1%	13086	73.9%	1441	64.6%	2218	67.3%	1138	73.8%
Missing	52012	25.9%	37740	23.4%	6420	26.6%	633	22.1%	4181	55.9%	3038	66.3%
*61–65*												
Dead	183	0.2%	166	0.2%	7	0.1%	2	0.2%	4	0.2%	4	0.4%
Critical	3314	3.2%	2889	3.2%	253	2.7%	59	4.4%	81	4.2%	32	2.8%
Emergent	26404	25.5%	23022	25.7%	2220	23.8%	354	26.7%	528	27.3%	280	24.9%
Low	73459	71.1%	63559	70.9%	6855	73.4%	913	68.8%	1322	68.3%	810	71.9%
Missing	35026	25.3%	26870	23.1%	3312	26.2%	335	20.1%	2471	56.1%	2038	64.4%
*66–70*												
Dead	156	0.1%	146	0.1%	5	0.1%	2	0.2%	2	0.1%	1	0.1%
Critical	3733	3.2%	3268	3.1%	271	3.5%	78	6.3%	84	4.3%	32	2.5%
Emergent	29925	25.6%	26894	25.7%	1891	24.4%	308	24.9%	567	29.1%	265	20.8%
Low	82986	71.0%	74271	71.0%	5593	72.1%	847	68.6%	1298	66.5%	977	76.6%
Missing	39316	25.2%	31603	23.2%	2804	26.5%	362	22.7%	2190	52.9%	2357	64.9%
*71–75*												
Dead	154	0.1%	140	0.1%	7	0.1%	3	0.3%	3	0.2%	1	0.1%
Critical	3979	3.0%	3617	2.9%	198	2.9%	69	5.9%	68	3.9%	27	1.8%
Emergent	34609	25.7%	31780	25.7%	1693	24.9%	339	29.1%	505	28.8%	292	20.0%
Low	96078	71.3%	88117	71.3%	4888	72.0%	752	64.7%	1178	67.2%	1143	78.1%
Missing	46366	25.6%	38286	23.6%	2550	27.3%	318	21.5%	2094	54.4%	3118	68.1%
*76–80*												
Dead	174	0.1%	167	0.1%	5	0.1%	1	0.1%	0	0.0%	1	0.1%
Critical	4147	2.8%	3836	2.8%	170	2.9%	45	4.8%	71	4.5%	25	1.5%
Emergent	37589	25.6%	34923	25.6%	1637	27.5%	265	28.1%	448	28.2%	316	18.9%
Low	104879	71.4%	97704	71.5%	4143	69.6%	633	67.1%	1071	67.4%	1328	79.5%
Missing	50954	25.8%	42686	23.8%	2231	27.3%	255	21.3%	1892	54.3%	3890	70.0%
Dead	174	0.1%	167	0.1%	5	0.1%	1	0.1%	0	0.0%	1	0.1%
*81–85*												
Dead	149	0.1%	141	0.1%	5	0.1%	0	0.0%	1	0.1%	2	0.1%
Critical	4144	2.6%	3884	2.6%	138	2.8%	34	5.2%	58	4.4%	30	1.6%
Emergent	39,227	24.9%	37019	24.9%	1318	26.8%	166	25.6%	330	25.2%	394	20.6%
Low	114226	72.4%	107,913	72.4%	3459	70.3%	448	69.1%	918	70.2%	1488	77.7%
Missing	55089	25.9%	46615	23.8%	1873	27.6%	173	21.1%	1851	58.6%	4577	70.5%
*86–90*												
Dead	142	0.1%	139	0.1%	2	0.1%	0	0.0%	0	0.0%	1	0.1%
Critical	3658	2.4%	3491	2.4%	102	3.0%	23	5.4%	21	2.2%	21	1.1%
Emergent	37176	24.3%	35490	24.3%	915	27.3%	136	32.1%	274	28.8%	361	18.5%
Low	111970	73.2%	107146	73.3%	2333	69.6%	265	62.5%	656	69.0%	1570	80.4%
Missing	53358	25.9%	45313	23.7%	1270	27.5%	93	18.0%	1515	61.4%	5167	72.6%
*91 + *												
Dead	120	0.1%	117	0.1%	1	0.1%	0	0.0%	0	0.0%	2	0.1%
Critical	3084	2.3%	2956	2.3%	54	3.1%	18	7.6%	23	4.0%	33	1.7%
Emergent	31846	23.7%	30788	23.7%	487	27.5%	72	30.4%	146	25.7%	353	18.4%
Low	99137	73.9%	95831	73.9%	1227	69.4%	147	62.0%	399	70.2%	1533	79.8%
Missing	46823	25.9%	39717	23.4%	724	29.0%	53	18.3%	1214	68.1%	5115	72.7%
Missing Age	5515	0.3%	4346	0.3%	733	0.6%	86	0.4%	109	0.2%	249	0.5%

**Table 5:** Descriptive Statistics for EMS Encounters for Reported Location of Fall Injuries by Need for Triage to a Trauma Center, Categorized by Age.^1^

	Overall		Indoor		Outdoor- Street or Sidewalk		Outdoor- Not on Street or Sidewalk		Indoor/outdoor unclear		Missing	
	*N* = 1854909		*N* = 1596860		*N* = 129408		*N* = 23586		*N* = 53700		*N* = 51355	
*0–20*												
Need for transport to Trauma Center	7231	8.4%	5854	9.0%	591	8.5%	223	4.4%	393	5.4%	170	9.0%
Does not	78754	91.6%	58994	91.0%	6353	91.5%	4840	95.6%	6855	94.6%	1712	91.0%
Missing	32154	27.2%	25481	28.2%	2054	22.8%	1772	25.9%	1813	20.0%	1034	35.5%
*21–30*												
Need for transport to Trauma Center	3588	5.8%	2397	5.5%	811	7.6%	86	4.9%	228	5.5%	66	4.8%
Does not	58079	94.2%	41339	94.5%	9881	92.4%	1656	95.1%	3,882	94.5%	1321	95.2%
Missing	9285	13.1%	6056	12.2%	1969	15.6%	393	18.4%	498	10.8%	369	21.0%
*31–40*												
Need for transport to Trauma Center	4417	6.2%	3152	6.0%	908	7.9%	82	4.8%	205	5.1%	70	4.7%
Does not	67245	93.8%	49813	94.0%	10596	92.1%	1637	95.2%	3781	94.9%	1418	95.3%
Missing	10347	12.6%	7145	11.9%	2028	15.0%	324	15.9%	441	10.0%	409	21.6%
*41–50*												
Need for transport to Trauma Center	5493	6.0%	4167	5.9%	886	7.0%	82	4.7%	269	6.3%	89	4.8%
Does not	86218	94.0%	67011	94.1%	11781	93.0%	1646	95.3%	4028	93.7%	1752	95.2%
Missing	12502	12.0%	9138	11.4%	2054	14.0%	329	16.0%	440	9.3%	541	22.7%
*51–60*												
Need for transport to Trauma Center	10231	5.8%	8262	5.8%	1305	6.2%	114	4.8%	372	5.4%	178	5.0%
Does not	166488	94.2%	134674	94.2%	19704	93.8%	2258	95.2%	6464	94.6%	3388	95.0%
Missing	23782	11.9%	18527	11.5%	3113	12.9%	490	17.1%	639	8.5%	1013	22.1%
*61–65*												
Need for transport to Trauma Center	6664	5.5%	5710	5.5%	573	5.2%	60	4.3%	208	5.1%	113	4.6%
Does not	115516	94.5%	97482	94.5%	10496	94.8%	1349	95.7%	3853	94.9%	2336	95.4%
Missing	16206	11.7%	13314	11.4%	1578	12.5%	254	15.3%	345	7.8%	715	22.6%
*66–70*												
Need for transport to Trauma Center	7073	5.1%	6303	5.2%	387	4.2%	58	4.3%	177	4.6%	148	5.3%
Does not	130439	94.9%	114077	94.8%	8781	95.8%	1300	95.7%	3633	95.4%	2648	94.7%
Missing	18604	11.9%	15802	11.6%	1396	13.2%	239	15.0%	331	8.0%	836	23.0%
*71–75*												
Need for transport to Trauma Center	7729	4.8%	7089	5.0%	308	3.8%	50	3.9%	145	4.1%	137	4.1%
Does not	151707	95.2%	135990	95.0%	7862	96.2%	1241	96.1%	3387	95.9%	3227	95.9%
Missing	21750	12.0%	18861	11.6%	1166	12.5%	190	12.8%	316	8.2%	1217	26.6%
*76–80*												
Need for transport to Trauma Center	8219	4.7%	7617	4.8%	262	3.7%	32	3.2%	135	4.3%	173	4.2%
Does not	165532	95.3%	150722	95.2%	6884	96.3%	983	96.8%	3040	95.7%	3903	95.8%
Missing	23992	12.1%	20977	11.7%	1040	12.7%	184	15.3%	307	8.8%	1484	26.7%
*81–85*												
Need for transport to Trauma Center	8200	4.4%	7723	4.4%	159	2.7%	23	3.3%	105	3.6%	190	3.9%
Does not	179791	95.6%	165930	95.6%	5765	97.3%	676	96.7%	2795	96.4%	4625	96.1%
Missing	24,844	11.7%	21919	11.2%	869	12.8%	122	14.9%	258	8.2%	1676	25.8%
*86–90*												
Need for transport to Trauma Center	7907	4.3%	7500	4.4%	127	3.1%	20	4.7%	90	4.0%	170	3.3%
Does not	174027	95.7%	162504	95.6%	3933	96.9%	410	95.3%	2179	96.0%	5001	96.7%
Missing	24,370	11.8%	21575	11.3%	562	12.2%	87	16.8%	197	8.0%	1949	27.4%
*91 + *												
Need for transport to Trauma Center	7095	4.4%	6707	4.5%	95	4.4%	15	6.3%	72	4.3%	206	4.0%
Does not	152599	95.6%	143729	95.5%	2082	95.6%	222	93.7%	1602	95.7%	4964	96.0%
Missing	21316	11.8%	18973	11.2%	316	12.7%	53	18.3%	108	6.1%	1866	26.5%
Missing Age	5515	0.3%	4346	0.3%	733	0.6%	86	0.4%	109	0.2%	241	0.5%

^1^ T-RTS, Revised Trauma Score for Triage

## Discussion

The majority of fall injuries to which EMS responded in 2019 occurred indoors, with the second largest category occurring outdoors on streets or sidewalks. However, the proportion of patients who had fall injuries rated as Emergent or Critical, had moderate or severe GCS scores and had a T-RTS score indicating the need for transport to a Trauma Center were similar across indoor and outdoor locations of falls. Given the large numbers of falls that occur indoors and among older persons, it is appropriate that falls prevention guidelines and recommendations have focused on these falls (Panel on Prevention of Falls in Older Persons 2011; American Geriatrics Society, British geriatrics society, and American academy of orthopaedic surgeons panel on falls prevention 2001). However, the comparable trauma severity of outdoor injurious falls compared to those that occur indoors and the greater severity of falls on streets and sidewalks among young and middle-aged patients suggests that additional public health attention is needed to identify modifiable outdoor environmental risk factors to prevent outdoor falls.

This study found that the proportion of severe outdoor falls on streets and sidewalks was higher among men compared to women. This finding may be explained by differences in age and physical activity status for men and women falling outside versus inside (Timsina et al. 2017; Kelsey et al. 2012; Duckham et al. 2013). Timsina et al. (2017) found that young and middle-age men were more likely to fall outside, whereas older females were more likely to fall inside. Young men were also most likely to fall while engaging in vigorous activity, and thus the potentially higher speed and impact of the activities at the time of falling may result in more serious fall injuries for this subgroup. An additional explanation is that men of all ages tend to consume more alcohol than women, and acute alcohol consumption is associated with greater risk of injurious falls by impacting balance control and cognition (Taylor et al. 2010). One study found that alcohol-related fall injury presentations to emergency departments (ED) were more prevalent among men and younger patients, and were more severe based on triage scale ratings and admissions to the ED compared to non-alcohol-related injuries (Woods et al. 2019). Future work in this area should examine the role of substance use in the severity of injurious falls by location to inform place-based intervention strategies and policies.

We also found that outdoor falls on streets or sidewalks had higher proportions for injury severity scores among young and middle-aged individuals compared to indoor falls among this age subgroup, but this pattern was reversed for older adults. This finding may also be explained by the evidence showing a higher proportion of alcohol involved falls among younger adults and greater severity of these falls, which could be occurring on streets or sidewalks near alcohol serving establishments or nightlife districts where alcohol consumption is common (Woods et al. 2019). Indoor falls may be more frequently severe among older adults due to a form of selection bias. Specifically, the population of older adults that fall indoors may be more likely to be frail, while those who fall outdoors may be in better overall health (Kelsey et al. 2012). This is consistent with findings that suggest that outdoor falls are experienced by healthier and more active individuals, compared to the greater risk of falling indoors for individuals in poorer health who may experience worse injury outcomes. (Li et al. 2006; Kelsey et al. 2010). Additionally, older adults often have medical conditions and are more likely to use medications such as psychotropic and cardiovascular drugs that increase their risk of falling (Seppala et al. 2018; Wastesson et al. 2018). Lastly, there may also be differences in the types of surfaces (e.g., wooden floor, grass) or floor characteristics adults are falling on indoors compared to outdoors which could influence injury severity; however, studies are lacking in this area of research (Jung et al. 2018).

Current fall prevention guidelines do not explicitly examine the impact of outdoor environments on falls (Panel on Prevention of Falls in Older Persons 2011; Montero-Odasso et al. 2022) and pedestrian safety policies are largely centered around pedestrian injuries from motor vehicles with minimal attention to outdoor falls, even though these two injury types occur in the same or adjacent physical environments (Evenson et al. 2018). This may be due to the limited empirical evidence available to determine modifiable environmental risk factors for outdoor falls (Schepers et al. 2017). Li et al. (2006) found that among a sample of U.S. adults, participants subjectively reported that most (73%) of outdoor falls were due to environmental factors such as the condition of the walking surface, and usually occurred on sidewalks, curbs and streets.Yet, rigorous epidemiological studies are still needed to identify potential environmental hazards on sidewalks and streets such as street trees that may cause buckling or damage to sidewalks and increase outdoor fall risk (Bentley 1998; Bentley and Haslam 2001; David and Freedman 1990; Fothergill et al. 1995; Hunt et al. 1991). The described approach using routinely collected EMS administrative and clinical data for surveillance of outdoor injurious falls could be used in future research studies that implement ecological or case–control study designs to identify risk factors for outdoor fall injuries or for evaluating interventions to reduce injurious falls (Mooney et al. 2022).

The primary strength of this study is the use of NEMSIS data, which provides a well-documented, very large census of health encounters requiring an EMS response. The data include pertinent sociodemographic and clinical information, and variables that can be used to code the location of the encounter, eliminating the need to incorporate additional data sources. While sensitivity analyses revealed that there were essentially no differences in presented findings when using the mean, first, max, or min GCS or T-RTS score, measurement error is still possible given the existing concerns regarding the accuracy and validity of the GCS (Bledsoe et al. 2015). The severity outcome data also had varying degrees of missingness. We were unable to calculate GCS for 7.5% of patients and T-RTS for 13.0% of patients, and 25.9% of patients did not have an acuity measure reported. Also, there are many ICD 10 sub-codes available for defining falls, but it is unclear how much variation there is across EMS clinicians and companies in coding falls by ICD 10 codes. As such we did not attempt to sub-classify falls by context, such as falls on stairs or falls involving impacts on furniture. This study is also limited by missing data on fall location, but only 6% of fall injuries could not be classified by location. The coding schema used to classify fall location does not make use of narratives and text notes created by EMS personnel, and therefore may result in misclassification of fall location. Machine learning for natural language processing applied to EMS narrative notes could supplement the ICD 10-based case-finding algorithm used in this study and increase the sensitivity of identifying fall location from EMS data (Mayampurath et al. 2021; Zhao et al. 2021).

Lastly, the NHIS estimate for the percentage of injurious outdoor falls is substantially higher than we observed in the NEMSIS data (47% vs 9%) (Timsina et al. 2017). NHIS includes injurious falls that required any medical attention and collects data from community dwelling adults, while NEMSIS provides data on EMS responses for all ages and for those living in the community and in nursing facilities. As outdoor falls have been found to occur among those who are younger and healthier (Li et al. 2006; Kelsey et al. 2010), these falls may be less likely to require an EMS response than those occurring indoors and suggests that a selection bias exists within the NEMSIS sample. As such the differences in the estimates of the proportions of falls occurring outdoors derived from the NHIS and NEMSIS likely reflect differences in the clinical thresholds used for defining an injurious fall and in the populations covered by the two datasets.

## Conclusion

In conclusion, these data show that the proportion of severe life-threatening injuries from falls that occur outdoors on streets or sidewalks is similar to that for falls that occur indoors. These findings represent a public concern as the population of persons age 65 years and older is expected to grow by 22% by 2040, and the number of injurious falls and associated healthcare costs will simultaneously increase (Administration for Community Living 2022). Indeed, recent data already shows a rising incidence of falls of 1.5% per year from 2016 to 2019 (Hoffman et al. 2022). These concerns emphasize the need to address outdoor falls in current fall prevention guidelines, and to improve surveillance tools for monitoring outdoor falls and associated risk factors and outcomes.

## Data Availability

The datasets analyzed during the current study are publicly available at https://nemsis.org.

## Abbreviations

BRFSS: Behavioral Risk Factor Surveillance System
EMS: Emergency Medical Services
WISQARS: Web-Based Injury Statistics Query and Reporting System
ICD 10: International Classification of Diseases 10th Revision
NHIS: National Health Interview Survey
NEMSIS: National Emergency Medical Services Information System
ISS: Injury severity score
GCS: Glasgow Coma Scale
T-RTS: Revised Trauma Score for Triage
ED: Emergency departments
NHTSA: National Highway Traffic Safety Administration
